# Quantum capacitance, electrostatic potential, electronic and structural data for bare and functionalized niobium carbide MXenes

**DOI:** 10.1016/j.dib.2017.10.003

**Published:** 2017-10-06

**Authors:** Yan Xin, Yang-Xin Yu

**Affiliations:** a Laboratory of Chemical Engineering Thermodynamics, Department of Chemical Engineering, Tsinghua University, Beijing 100084, PR China; b State Key Laboratory of Chemical Engineering, Department of Chemical Engineering, Tsinghua University, Beijing 100084, PR China

## Abstract

The data reported in this article are structural and physicochemical properties for bare and F, O, OH and CH_3_O-functionalized Nb_*n*+1_C_*n*_ (*n* = 1, 2, 3 and 4) MXenes. The structural properties are presented as top views and side views from the X direction of the optimal structures of studied MXenes. The physicochemical properties include quantum capacitances, electrostatic potentials and electronic properties such as the projected density of states (PDOS) and band structures. Further interpretation and discussion of these data can be obtained from the article entitled “Possibility of bare and functionalized niobium carbide MXenes for electrode materials of supercapacitors and field emitters” (Xin and Yu, 2017) [1].

**Specifications Table**

**Table:** 

Subject area	*Electronic and energy engineering*
More specific subject area	*Electrode materials of supercapacitors; Materials for field emitters*
Type of data	*Table and figure*
How data was acquired	Software: Cambridge Sequential Total Energy Package (CASTEP) in Materials Studios 6.0 by Accelrys Software Inc.
	Electronic computers: Intel(R) Core™ i7 CPU 5930k
Data format	*Analyzed*
Experimental factors	*No pretreatment of samples was performed*
Experimental features	*Structural data obtained after geometry optimization completed successfully*
	*Electronic property and electrostatic potential data gathered by Dmol*^*3*^*analysis*
	*Quantum capacitance data gathered by numerical integration of density of states*
Data source location	*Beijing, P. R. China*
Data accessibility	*Data are with this article*

**Value of the data**

• The electrostatic potential data could serve as a benchmark for the researchers in designing the electrodes of field emitters.
• The quantum capacitance data could be used to select the materials for cathode and anode of supercapacitors.
• The projected density of states (PDOS) and band structures would provide a reference data for designers of electronics.

## 1 Data

The data in this article have been gathered under a program to study physicochemical properties of MXenes as energy and electronics materials.

**Fig. 1:**
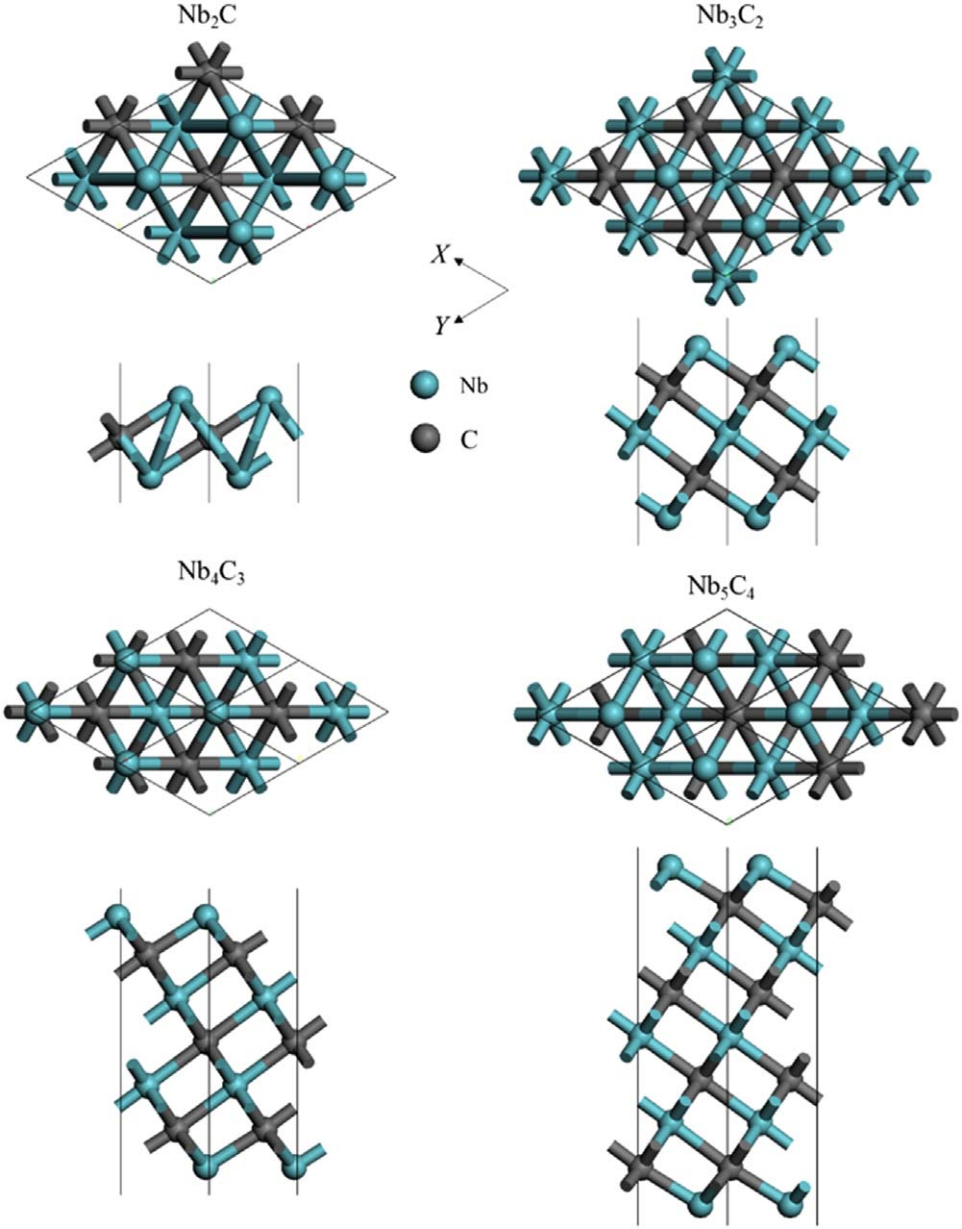
Top views and side views from the X direction of the optimal structures of Nb_2_C, Nb_3_C_2_, Nb_4_C_3_ and Nb_5_C_4_.

**Fig. 2:**
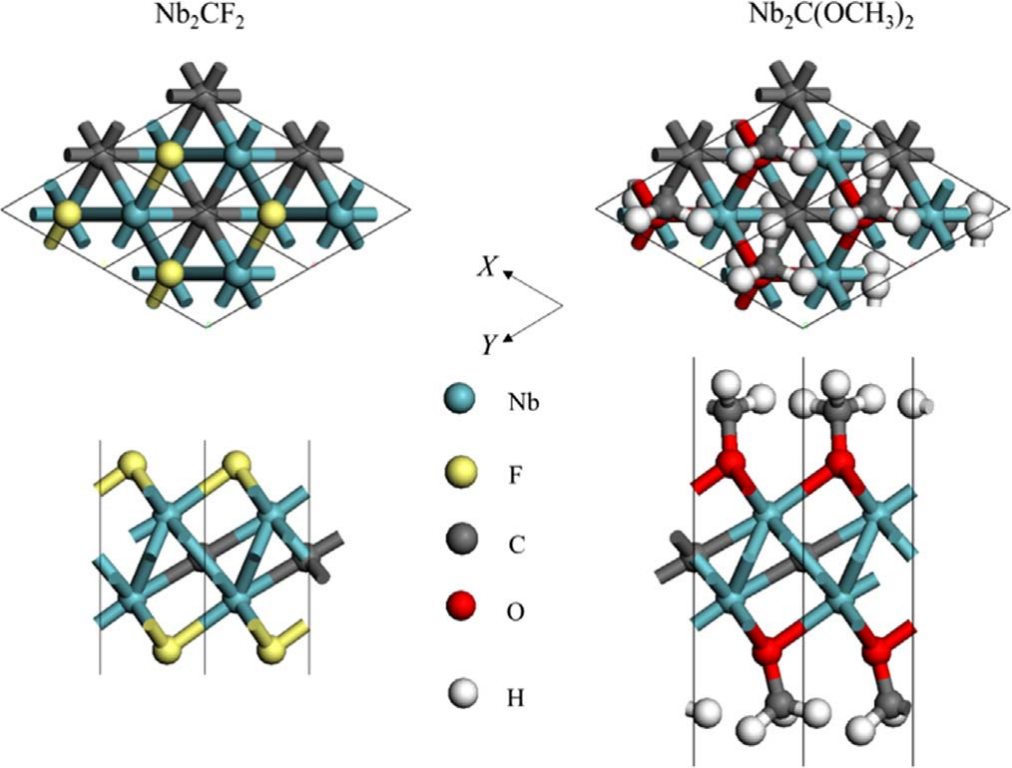
Top views and side views from the X direction of the optimal structures of Nb_2_CF_2_ and Nb_2_C(OCH_3_)_2_.

**Fig. 3:**
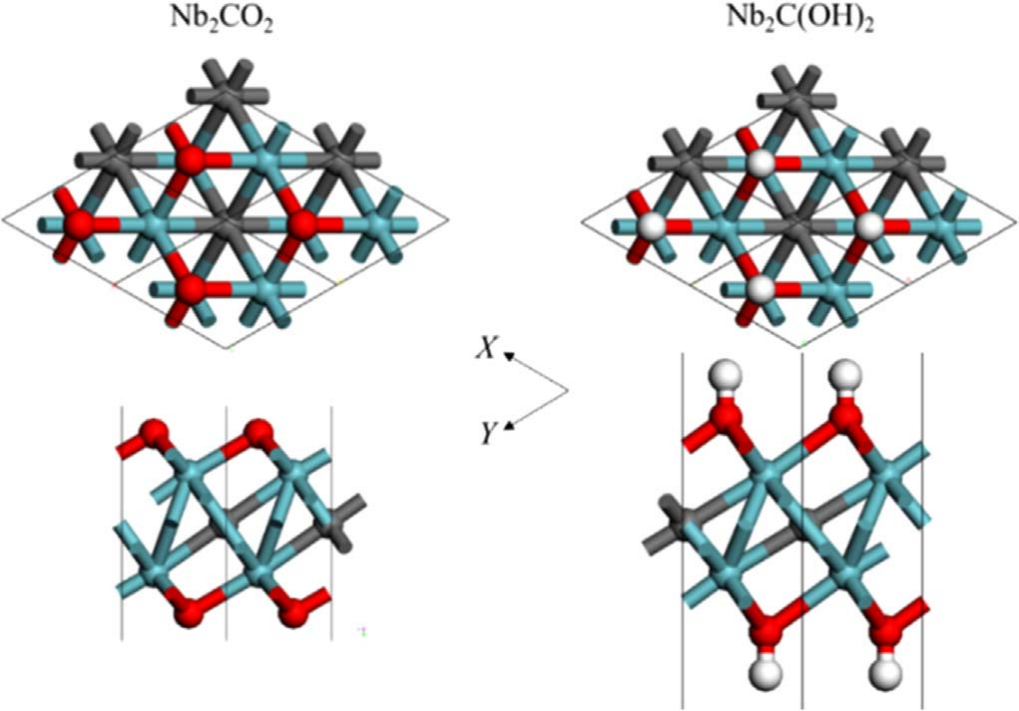
Top views and side views from the X direction of the optimal structures of Nb_2_CO_2_ and Nb_2_C(OH)_2_. The atoms represented by different color balls are the same as in Fig. 2.

**Fig. 4:**
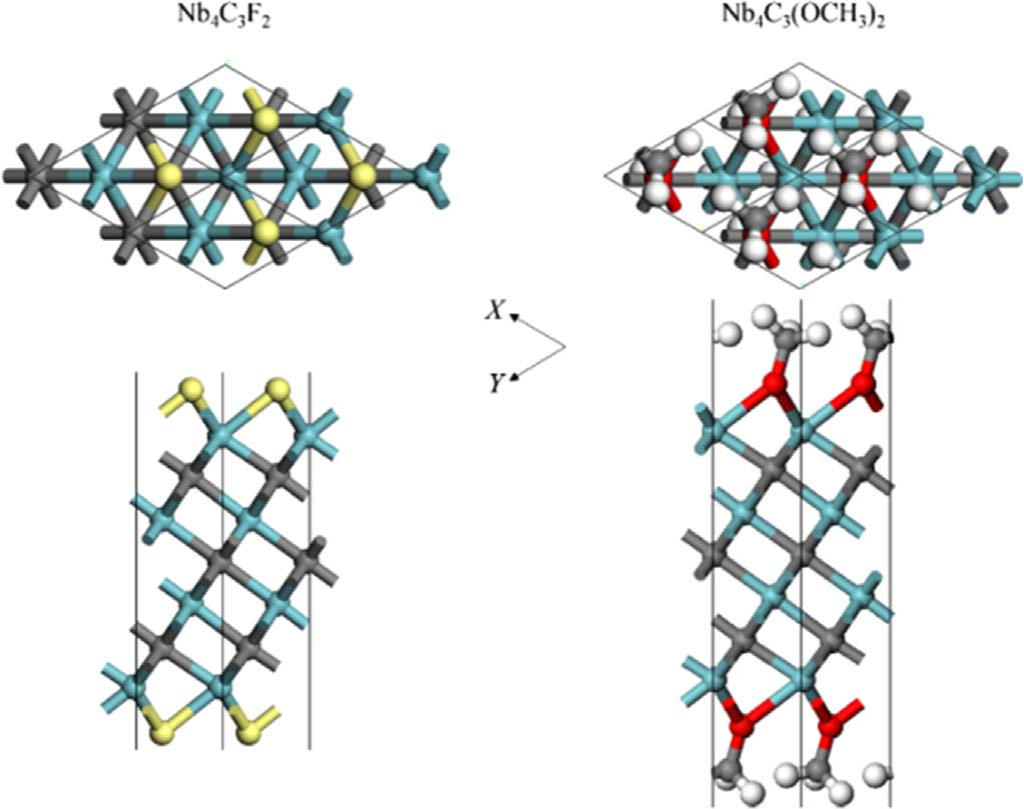
Top views and side views from the X direction of the optimal structures of Nb_4_C_3_F_2_ and Nb_4_C_3_(OCH_3_)_2_. The atoms represented by different color balls are the same as in Fig. 2.

**Fig. 5:**
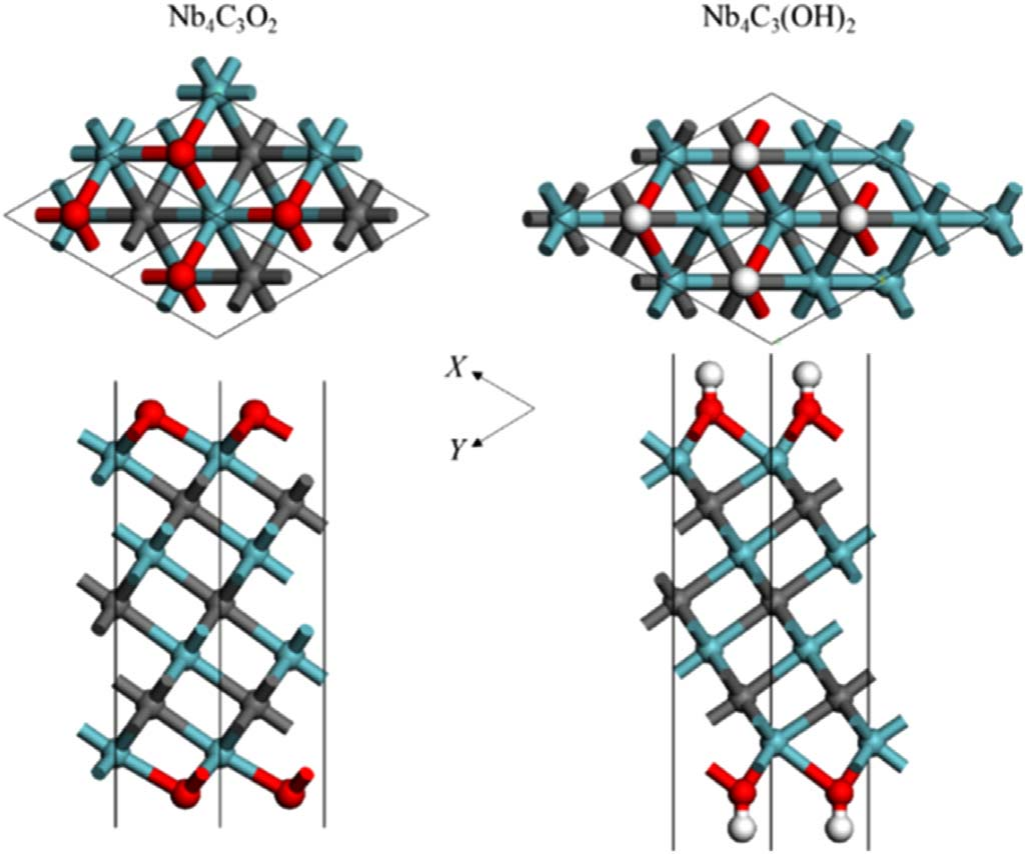
Top views and side views from the X direction of the optimal structures of Nb_4_C_3_O_2_ and Nb_4_C_3_(OH)_2_. The atoms represented by different color balls are the same as in Fig. 2.

**Fig. 6:**
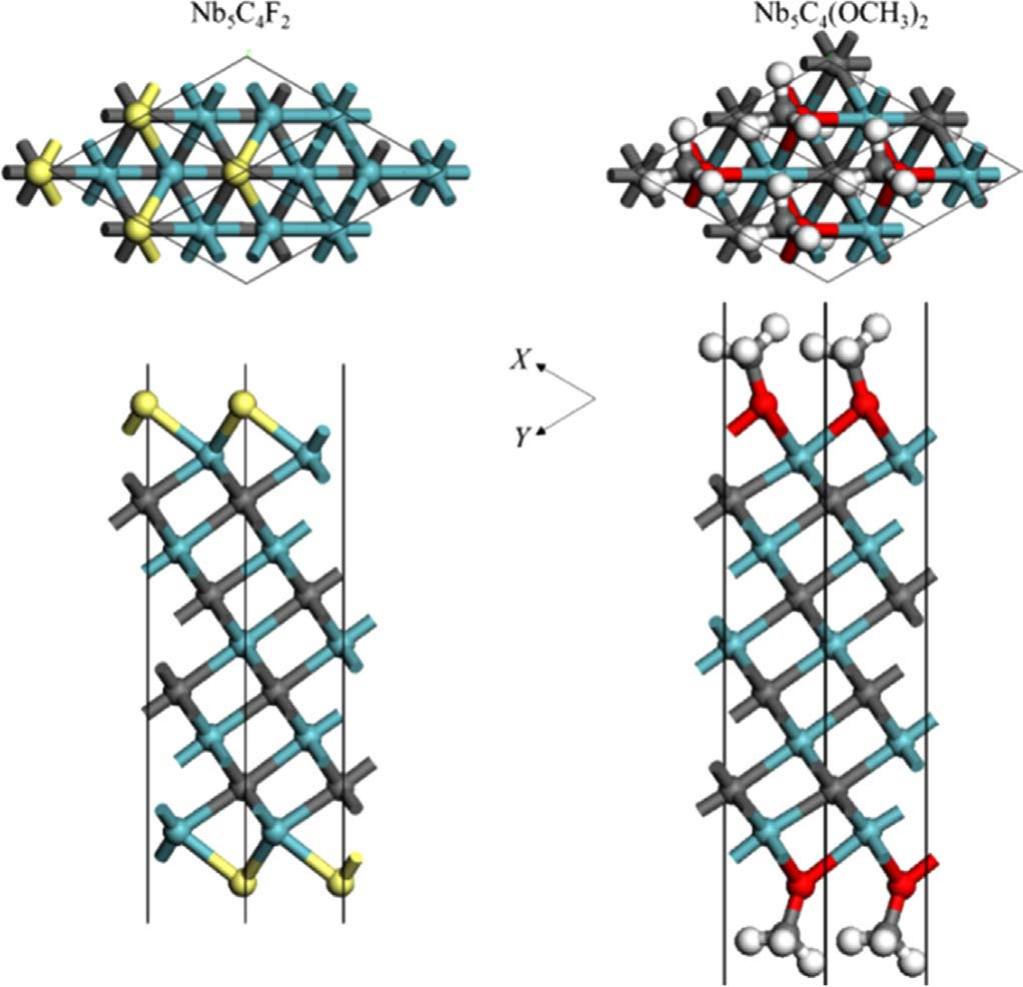
Top views and side views from the X direction of the optimal structures of Nb_5_C_4_F_2_ and Nb_5_C_4_(OCH_3_)_2_. The atoms represented by different color balls are the same as in Fig. 2.

**Fig. 7:**
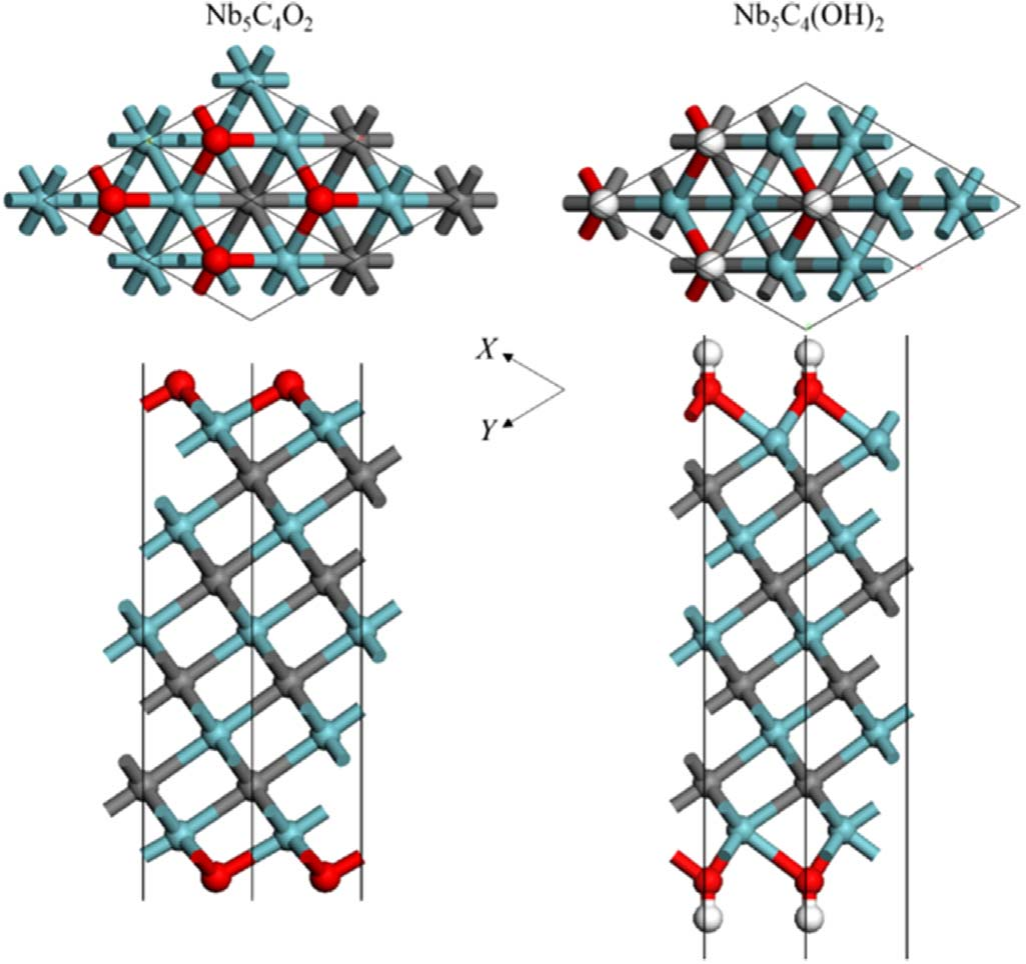
Top views and side views from the X direction of the optimal structures of Nb_5_C_4_O_2_ and Nb_5_C_4_(OH)_2_. The atoms represented by different color balls are the same as in Fig. 2.

**Fig. 8:**
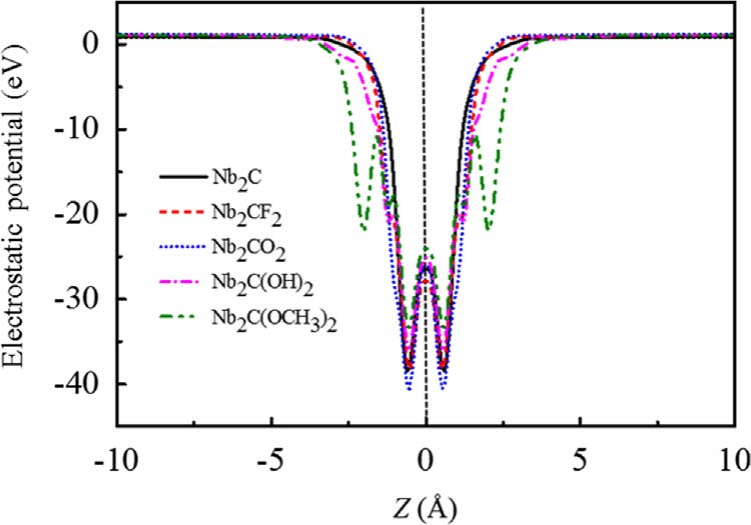
Planar averaged electrostatic potentials along the *z*-direction of Nb_2_C and Nb_2_CZ_2_ (Z = F, O, OH, OCH_3_). The peaks in green circles are enlarged to be more clearly.

**Fig. 9:**
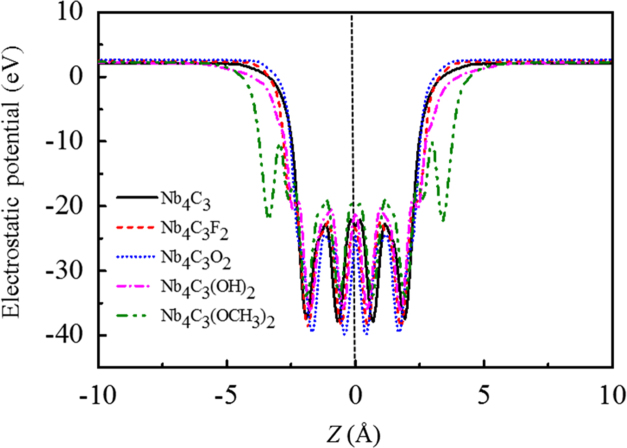
Planar averaged electrostatic potentials along the Z direction of Nb_4_C_3_ and Nb_4_C_3_Z_2_ (Z = F, O, OH, OCH_3_). The peaks in green circles are enlarged to be more clearly.

**Fig. 10:**
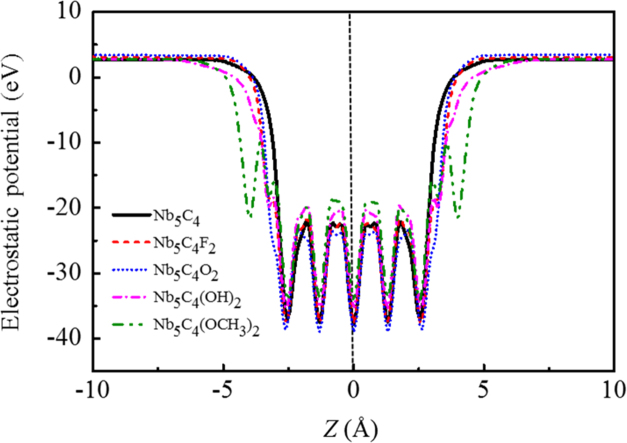
Planar averaged electrostatic potentials along the Z direction of Nb_5_C_4_ and Nb_5_C_4_Z_2_ (Z = F, O, OH, OCH_3_). The peaks in green circles are enlarged to be more clearly.

**Fig. 11:**
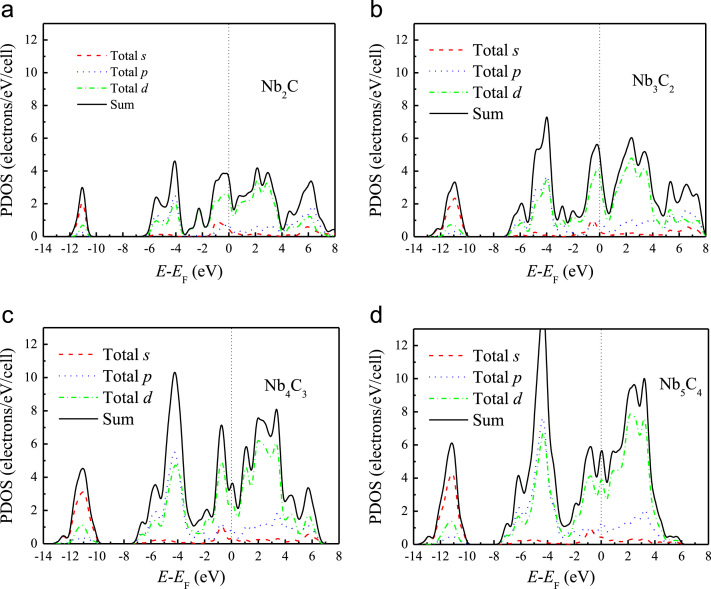
The PDOS of (a) Nb_2_C, (b) Nb_3_C_2_, (c) Nb_4_C_3_ and (d) Nb_5_C_4_.

**Fig. 12:**
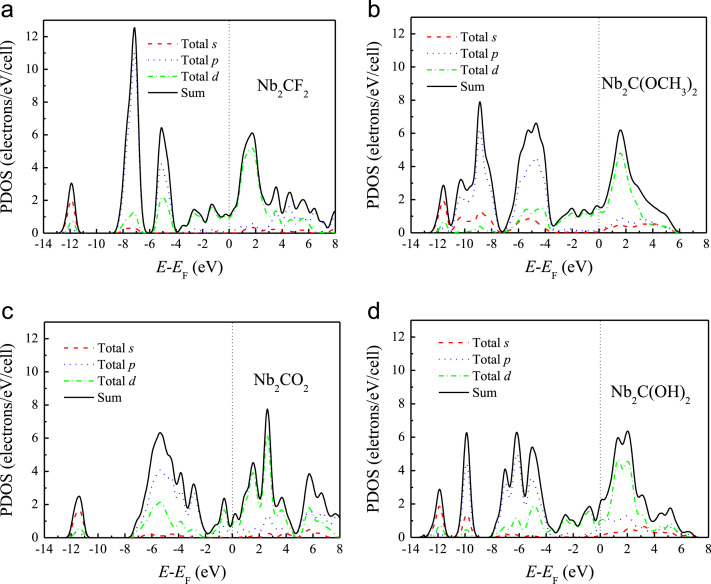
The PDOS of (a) Nb_2_CF_2_, (b) Nb_2_C(OCH_3_)_2_, (c) Nb_2_CO_2_ and (d) Nb_2_C(OH)_2_.

**Fig. 13:**
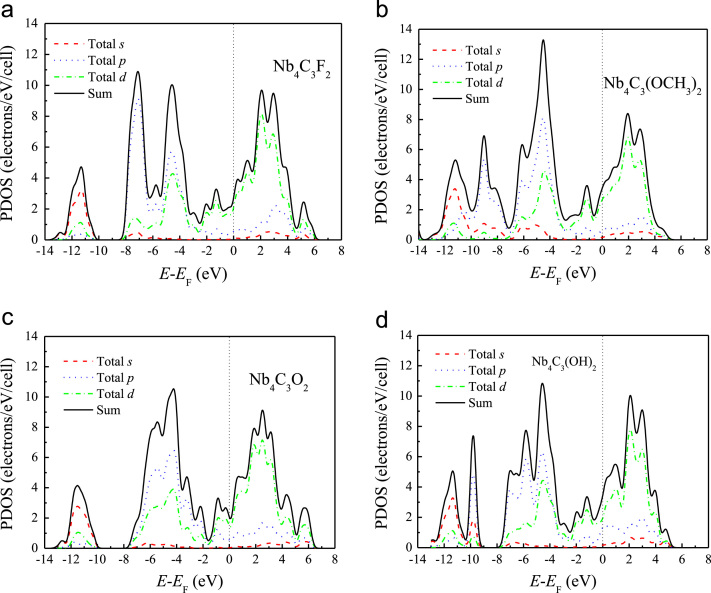
The PDOS of (a) Nb_4_C_3_F_2_, (b) Nb_4_C_3_(OCH_3_)_2_, (c) Nb_4_C_3_O_2_, and (d) Nb_4_C_3_(OH)_2_.

**Fig. 14:**
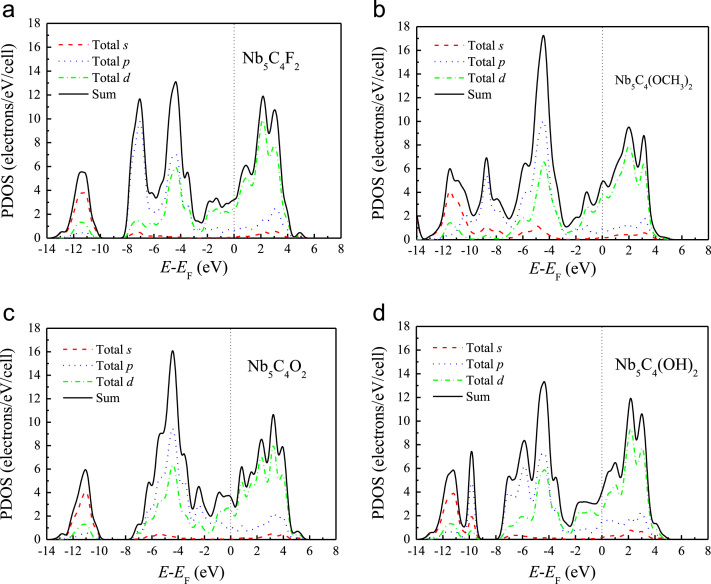
The PDOS of (a) Nb_5_C_4_F_2_, (b) Nb_5_C_4_(OCH_3_)_2_, (c) Nb_5_C_4_O_2_ and (d) Nb_5_C_4_(OH)_2_.

**Fig. 15:**
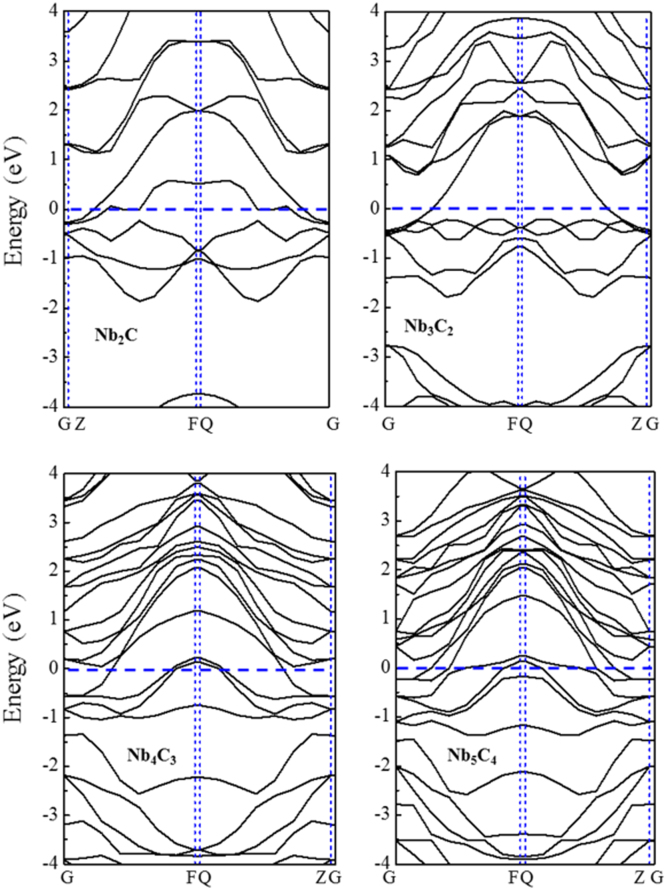
Band structures of the bare Nb_*n*+1_C_*n*_ (*n* = 1, 2, 3, 4).

**Fig. 16:**
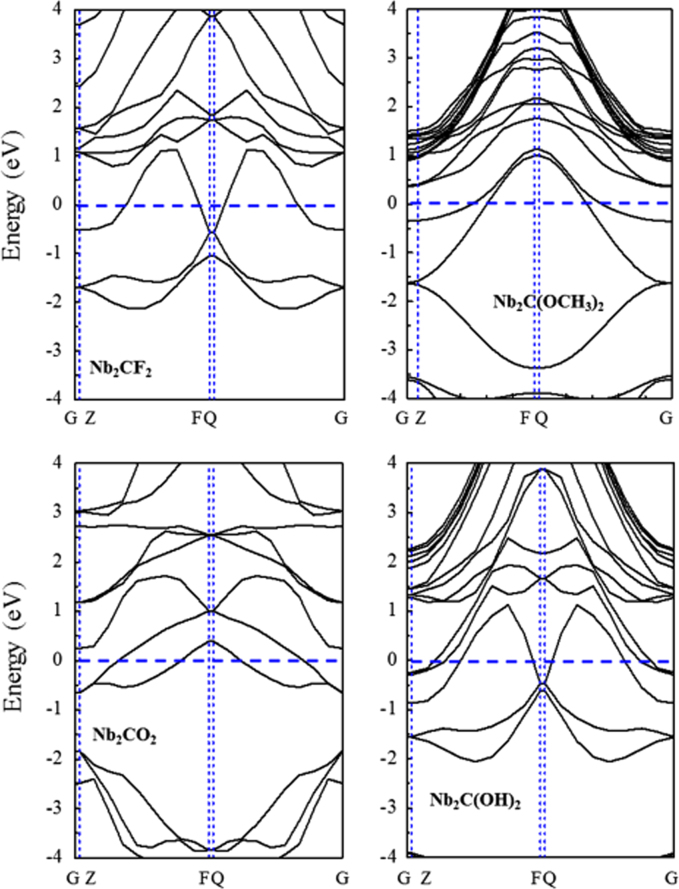
Band structures of Nb_2_CZ_2_ (Z = F, O, OH, OCH_3_).

**Fig. 17:**
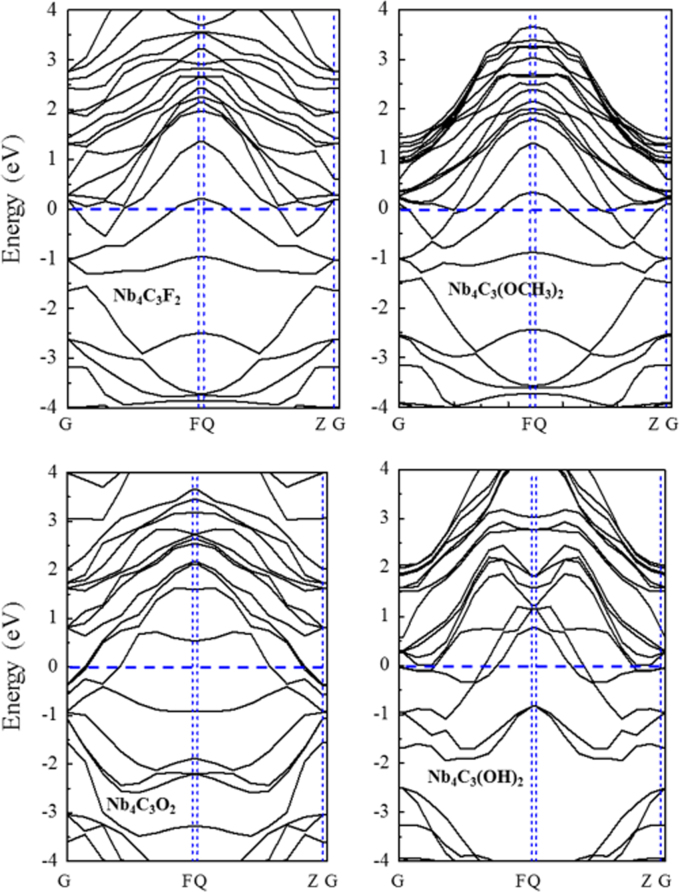
Band structures of Nb_4_C_3_Z_2_ (Z = F, O, OH, OCH_3_).

**Fig. 18:**
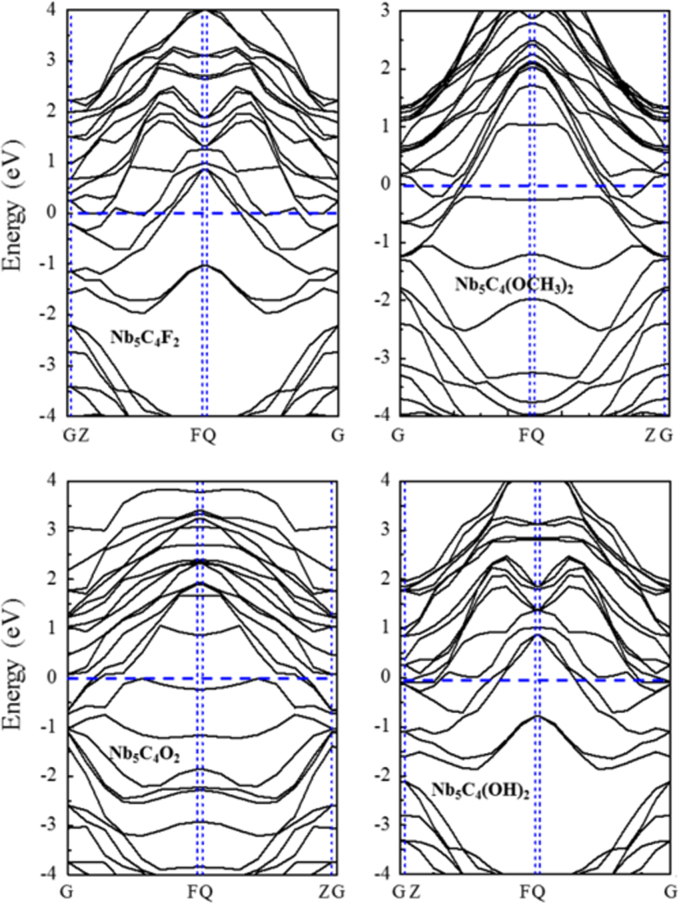
Band structures of Nb_5_C_4_Z_2_ (Z = F, O, OH, OCH_3_).

Fig. 1, Fig. 2, Fig. 3, Fig. 4, Fig. 5, Fig. 6, Fig. 7 demonstrate the top views and side views from the X direction of the optimal structures of Nb_*n*+1_C_*n*_ (*n* = 1, 2, 3 and 4), Nb_2_CF_2_ and Nb_2_C(OCH_3_)_2_, Nb_2_CO_2_ and Nb_2_C(OH)_2_, Nb_4_C_3_F_2_ and Nb_4_C_3_(OCH_3_)_2_, Nb_4_C_3_O_2_ and Nb_4_C_3_(OH)_2_, Nb_5_C_4_F_2_ and Nb_5_C_4_(OCH_3_)_2_ and Nb_5_C_4_O_2_ and Nb_5_C_4_(OH)_2_, respectively. Fig. 8, Fig. 9, Fig. 10 present the planar averaged electrostatic potentials along the Z direction of bare and functionalized Nb_*n*+1_C_*n*_Z_2_ (Z = F, O, OH, OCH_3_) for *n* = 1, 3 and 4, respectively. Fig. 11 depicts the PDOS of bare Nb_*n*+1_C_*n*_ (*n* = 1, 2, 3 and 4) MXenes. Fig. 12, Fig. 13, Fig. 14 depict the PDOS of functionalized Nb_*n*+1_C_*n*_Z_2_ (Z = F, O, OH, OCH_3_) for *n* = 1, 3 and 4, respectively. Fig. 15 presents the band structures of bare Nb_*n*+1_C_*n*_ (*n* = 1, 2, 3 and 4) MXenes. Fig. 16, Fig. 17, Fig. 18 are band structures of functionalized Nb_*n*+1_C_*n*_Z_2_ (Z = F, O, OH, OCH_3_) for *n* = 1, 3 and 4, respectively. Table 1 shows the values of the quantum capacitance (*C*_Q_ in *μ*F/cm^2^) of bare and F, O, OH and CH_3_O-functionalized Nb_*n*+1_C_*n*_ (*n* = 1, 2, 3 and 4) MXenes.

**Table 1:** The quantum capacitance *C*_*Q*_ of Nb_*n*+1_C_*n*_X_2_ (*n* = 1, 2, 3, 4, X = F, O, OH, and OCH_3_) at the negative and positive electrodes.

MXenes	*C*_*Q*_ (μF/cm^2^), *ϕ*_*G*_ = −0.62	*C*_*Q*_ (μF/cm^2^), *ϕ*_*G*_ = 0.83
Nb_2_C	211.2	354.0
Nb_2_CF_2_	128.2	96.8
Nb_2_CO_2_	119.7	144.1
Nb_2_C(OH)_2_	214.6	134.0
Nb_2_C(OCH_3_)_2_	147.6	117.9
Nb_3_C_2_	287.8	497.3
Nb_3_C_2_F_2_	220.8	161.4
Nb_3_C_2_O_2_	297.6	159.4
Nb_3_C_2_(OH)_2_	271.4	202.1
Nb_3_C_2_(OCH_3_)_2_	257.3	173.3
Nb_4_C_3_	267.3	475.0
Nb_4_C_3_F_2_	352.1	202.1
Nb_4_C_3_O_2_	294.2	247.1
Nb_4_C_3_(OH)_2_	422.5	224.5
Nb_4_C_3_(OCH_3_)_2_	363.0	215.0
Nb_5_C_4_	433.0	472.1
Nb_5_C_4_F_2_	396.5	282.4
Nb_5_C_4_O_2_	314.6	355.5
Nb_5_C_4_(OH)_2_	485.8	307.5
Nb_5_C_4_(OCH_3_)_2_	435.6	336.1

## 2 Computational methods

The figures are the outcome of the CASTEP and the quantum capacitance data were calculated using numerical integration of DOS. More details can be found in [1].
